# Recent sequence variation in probe binding site affected detection of respiratory syncytial virus group B by real-time RT-PCR

**DOI:** 10.1016/j.jcv.2016.12.011

**Published:** 2017-03

**Authors:** Everlyn Kamau, Charles N. Agoti, Clement S. Lewa, John Oketch, Betty E. Owor, Grieven P. Otieno, Anne Bett, Patricia A. Cane, D. James Nokes

**Affiliations:** a Epidemiology and Demography Department, Kenya Medical Research Institute (KEMRI) – Wellcome Trust Research Programme, Kilifi, Kenya; b School of Health and Human Sciences, Pwani University, Kilifi, Kenya; c Public Health England, Salisbury, United Kingdom; d School of Life Sciences and WIDER, University of Warwick, Coventry, United Kingdom

**Keywords:** RSV, Real-time, RT-PCR, Primer, Probe, Mismatches

## Abstract

**Background:**

Direct immuno-fluorescence test (IFAT) and multiplex real-time RT-PCR have been central to RSV diagnosis in Kilifi, Kenya. Recently, these two methods showed discrepancies with an increasing number of PCR undetectable RSV-B viruses.

**Objectives:**

Establish if mismatches in the primer and probe binding sites could have reduced real-time RT-PCR sensitivity.

**Study design:**

Nucleoprotein (N) and glycoprotein (G) genes were sequenced for real-time RT-PCR positive and negative samples. Primer and probe binding regions in N gene were checked for mismatches and phylogenetic analyses done to determine molecular epidemiology of these viruses. New primers and probe were designed and tested on the previously real-time RT-PCR negative samples.

**Results:**

N gene sequences revealed 3 different mismatches in the probe target site of PCR negative, IFAT positive viruses. The primers target sites had no mismatches. Phylogenetic analysis of N and G genes showed that real-time RT-PCR positive and negative samples fell into distinct clades. Newly designed primers-probe pair improved detection and recovered previous PCR undetectable viruses.

**Conclusions:**

An emerging RSV-B variant is undetectable by a quite widely used real-time RT-PCR assay due to polymorphisms that influence probe hybridization affecting PCR accuracy.

## Highlights

• Sequence variation at probe target site inhibited detection of a new RSV-B variant.
• RSV-B virus diversity was consistent with real-time RT-PCR sensitivity.
• Reduced PCR insensitivity could underestimate disease prevalence in clinical settings.
• Regular check of primer and probe target sites for rapidly evolving viruses is key.

## 1 Background

Respiratory syncytial virus (RSV) is a major cause of seasonal epidemics of acute respiratory infections and hospitalization of infants and young children worldwide [1], [2], [3]. The RSV genome is a non-segmented, single-stranded, negative sense RNA molecule that encodes 11 proteins. Two antigenic groups exist (A and B) and are further classified into genotypes according to genetic variability within the attachment glycoprotein (G) gene [4]. In Kilifi, coastal Kenya, RSV epidemics are known to occur annually as recorded through hospital case surveillance [5]. In 2002, longitudinal surveillance was initiated at the Kilifi County Hospital (KCH), to improve understanding on RSV disease burden and epidemiology [5].

RSV diagnosis in pediatric admissions at KCH has throughout included a direct immuno-fluorescence test (IFAT) (RSV DFA kit, Light Diagnostics™), and in 2008, a custom multiplex real-time reverse transcription-PCR (rRT-PCR) [6], [7] was implemented to screen for a range of respiratory viruses and for improved diagnostic sensitivity. The rRT-PCR employs RSV A and B specific primers and probes that target the nucleoprotein (N) gene in a triplex reaction including adenovirus. Nucleic acid (NA) amplification techniques have been widely adopted for detection of viruses [8]. However, false negatives can arise due to mismatches in primers or probes due to new polymorphisms in viral genomes [9] leading to subsequent underestimation of disease burden, unclear epidemiology and misguided clinical management. We observed a discordance of RSV-B detection between IFAT and rRT-PCR methods in the recent seasonal epidemics (2014/15 and 2015/16) and postulated that this was due to probe-template nucleotide mismatches.

## 2 Objective

We set out to examine the primer and probe binding regions to determine how the viruses that were detected by both IFAT and rRT-PCR methods differed from those detectable by IFAT but not by rRT-PCR.

### 2.1 Study design

Nasal swabs were obtained from pediatric patients admitted with acute respiratory illness. The Scientific and Ethical Review Unit, KEMRI, Kenya and the Coventry Research Ethics Committee, UK approved the study protocols for the surveillance and informed consent was sought accordingly. All samples (n = 1251) were tested by IFAT and total RNA, for the IFAT positive samples (n = 258, 20.62%), was extracted by RNeasy 96 QIAcube HT kit (Qiagen) using 140 μl of the swab sample, followed by one-step multiplex rRT-PCR (Qiagen, ABI 7500 system). IFAT is used as the gold standard assay for RSV detection. The RSV-B specific primers and probe (Old N assay) used are listed in Table 1, and our multiplex rRT-PCR has been described previously [7].

Fast-track Diagnostics® (FTD) FLU/HRSV rRT-PCR commercial kit and published primers and probe [10] were used as alternative assays to detect RSV in samples that gave discordant results between IFAT and the custom (in-house) multiplex rRT-PCR. Both alternative assays target the RSV matrix gene, different from our assay, which targets the nucleoprotein gene.

G and N genes were amplified by one-step RT-PCR (Qiagen) and sequenced (ABI 3700) for all samples that were IFAT positive. G gene amplification PCR is described in [11] and the N gene primers are listed in Table 1. The latter were designed to flank the RSV-B rRT-PCR primer and probe annealing sites. N and G gene sequences were assembled using Sequencher v5.0 (Gene Code Corp., USA). RSV-B rRT-PCR primers and probe sequences were aligned with the N gene sequences in a custom BLAST search to check for mismatching using *geneious* v.8.1.8 [12]. Phylogenetic analyses for N (700 bp) and G (905 bp) sequences were done by maximum likelihood method in MEGA v6.0 [13]. All sequences reported here are deposited in Genbank (KX775772-KX775940).

## 3 Results

**Fig. 1:**
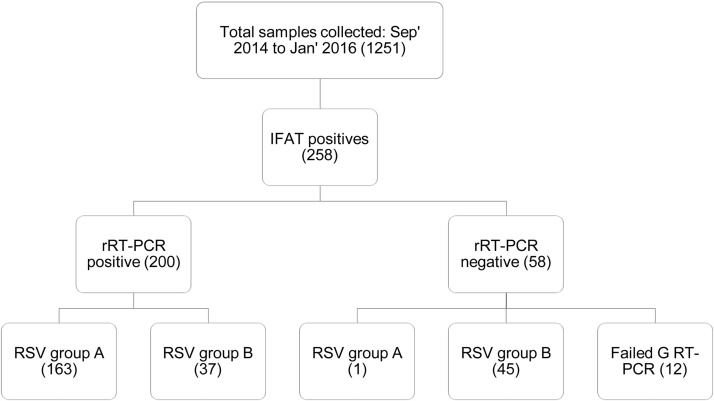
Summary of samples and rRT-PCR sensitivity for two RSV epidemics in Kilifi, Coastal Kenya 2014–2016.

Fifty-eight samples (22.5%) positive for RSV by the IFAT method were negative by rRT-PCR (threshold cycle (*C_T_*) value >35.0). RT-PCR products were obtained for N and G genes as revealed by gel electrophoresis, despite the lack of detection by real-time RT-PCR. G gene RT-PCR was successful for 46 of the discordant 58 samples: 45 were RSV-B genotype BA, 1 was RSV group A (Fig. 1). Moreover, the discordant samples re-tested RSV positive by FTD (36/58, 62.1%) and by the Pretorius assay [13] (45/58, 77.6%), suggesting a relative insensitivity with our custom RSV-B rRT-PCR.

**Fig. 2:**
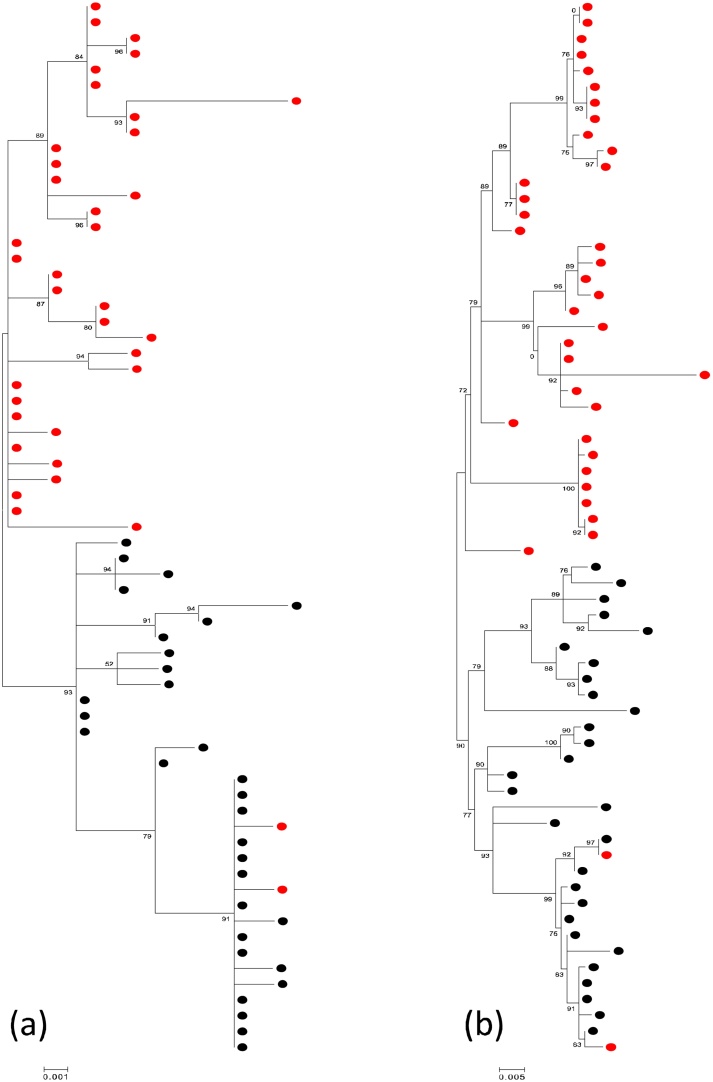
Phylogenetic clustering of nucleotide sequences from viruses undetectable and detectable by multiplex rRT-PCR from Kilifi, Coastal Kenya 2014–16. (a) Maximum likelihood tree of 72 N gene (700 bp) sequences: tips are colored by rRT-PCR outcome, where red is rRT-PCR negative (n = 40) and black is rRT-PCR positive (n = 32). (b) Maximum likelihood tree of 66 G gene (905 bp) sequences. Tips are colored as in (a). Viruses undetected by rRT-PCR formed a cluster separate from the detectable viruses sampled in the same epidemic. N and G gene sequences are derived from the same samples (matching sequences). The two RT-PCR negative strains had an additional polymorphism as described in text.

Phylogenetic analyses of the matching N and G gene sequences showed that the rRT-PCR negative RSV-B viruses are a separate phylogenetic clade distinct from the positive viruses (Fig. 2). Comparing the G gene sequences to other RSV-B viruses sampled in the affected (2014/15 and 2015/16) and previous epidemics, the rRT-PCR negative strains formed a separate phylogenetic group. Similarly, this distinct clustering was seen when comparing the rRT-PCR negative viruses with contemporaneous global genotype BA G gene data (analysis not shown here).

**Fig. 3:**
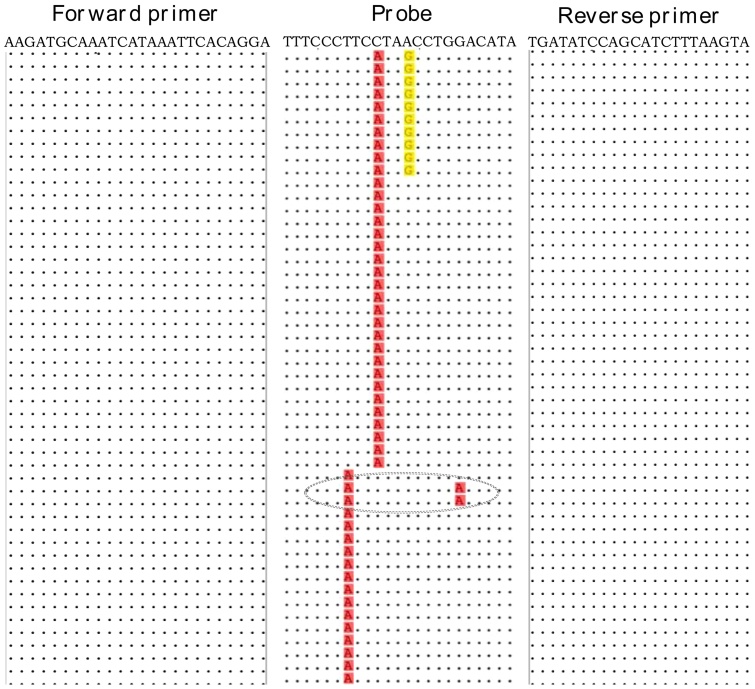
An alignment (5‘ – 3‘) of the primers and probe with a subset of N gene sequences from the rRT-PCR discrepant samples: dots indicate identity with primer or probe sequence. Mismatching nucleotides at positions 11, 14 and 17 of the probe are highlighted. Majority of the rRT-PCR negative RSV-B viruses had nucleotide changes at the 14th base. The grey circle shows the additional polymorphism at the 6th base for two PCR negative strains that clustered with rRT-PCR positive viruses.

Examining the N gene sequences showed no mismatches in the forward and reverse primer binding sites for either rRT-PCR positive or negative RSV-B viruses. Conversely, three mismatches at positions 11 (T/G), 14 (C/A) and 17 (A/G) at the probe target site were unique to the rRT-PCR negative viruses (Fig. 3). Majority of the rRT-PCR negatives (27/45, 60%) had a mismatch at the 14th base; 10/45 (22%) had mismatches at the 11th and 14th bases; and 18/45 (40%) had a mismatch at the 17th base. Two other rRT-PCR negatives had a mutation at the 6th base (C/T) in addition to a mutation at the 17th base (gray circle, Fig. 3), and these clustered with the rRT-PCR positives for both N and G gene sequences (Fig. 2). None of the rRT-PCR negatives contained more than three mismatches. All RSV-B viruses, rRT-PCR positive and negative, had a mismatch at the 1st base (T/G) in the probe site implying the PCR conditions likely tolerated this mismatch.

We designed new primers and probe upstream of the old target site within N gene (Table 1) and tested them on the 58 IFAT positive, rRT-PCR negative samples, while keeping former rRT-PCR setup and reagent concentrations constant. The new N primers/probe detected 45/58 (77.6%) of the previously undetected viruses with mean *C_T_* value of 27.1 (range 19.8–34.6). The new primers and probe were also specific to RSV-B in presence of other respiratory viruses and have been shown to detect RSV-B in both singleplex and multiplex reactions.

## 4 Discussion

Sensitive detection of RSV is important for molecular epidemiology analyses and for tracking virus transmission. Our RSV-B rRT-PCR primers and probe was adopted from Gunson et al. [6] and some laboratories [14], [15], [16], [17] have recently reported using these particular primers and probe. Here, we report three point mutations in the probe hybridization site, which distinguished rRT-PCR negative and positive RSV-B viruses and likely reduced the probe binding efficiency. The mismatches were located in the middle of the probe. All viruses contained a purine-pyrimidine mismatch at the 1st base with likely minor impact on PCR performance. Nonetheless, it was possible to obtain RT-PCR products (N and G gene) from the same discrepant samples, an indication that the mutations in the probe-template binding site had a likely effect on rRT-PCR performance.

Newly designed N gene rRT-PCR primers and probe effectively detected the novel RSV-B variant in addition to other circulating RSV-B variants without decreasing assay specificity. Furthermore, re-testing using a commercial and previously published RSV rRT-PCR assays showed positive results; however, these alternative assays do not distinguish RSV-A and RSV-B and they target the matrix gene.

Based on phylogenetic analyses, rRT-PCR negative viruses genetically clustered separately from the detectable RSV-B viruses and the clustering does not appear to be random. Our results extend earlier reports and observations [18], [19], [20] on the influence of sequence variation and primer/probe mismatches on rRT-PCR performance. It is now unusual in routine diagnostic settings to maintain a front-line antigen detection method for diagnosis of respiratory viruses, and instead most settings just use molecular methods. Given the high prevalence of the RSV-B variant with the discordant results, continued use of the Gunson [6] RSV-B assay unaccompanied by an antigen detection test might broadly underestimate RSV prevalence. Our sustained use of IFAT has allowed us to detect a problem with a quite widely used set of primers and probes and highlights the importance of regular reviews of primer and probe binding sites, particularly for the rapidly evolving RNA viruses.

## Funding

This work was funded by the Wellcome Trust (grant ref: 102975).

## Ethical approval

The Scientific and Ethical Review Unit, KEMRI, Kenya and the Coventry Research Ethics Committee, UK approved the study protocols for the surveillance and informed consent was sought accordingly.

## Competing interests

None declared.

## Figures and Tables

**Table 1:** The primer and probe sequences for the old and new N rRT-PCR assays; forward and reverse primers for N gene RT-PCR. Mismatching positions in the probe sequence reported in the text are shown in bold and underlined.

Assay	Forward primer (5′-3′)	Reverse primer (5′-3′)	Probe sequence (5′-3′)
Old N*	AAGATGCAAATCATAAATTCACAGGA (1248–1273)^#^	TGATATCCAGCATCTTTAAGTA (1350–1329)^#^	VIC-TTTCCC**T**TC**C**TA**A**CCTG**G**ACAT**A**-TAMRA (1317–1294)^#^

New N**	GCATCATTGCTGTCATTAACTCAATT (2009–2034)^#^	GGTGTACCTCTRTACTCTCCCATTATG (2045–2070)^#^	VIC-TCAAGTGTGGTCYTAGGYAATGCAGC-TAMRA (2107–2080)^#^

N gene RT-PCR	GCAAATAYAAARATGGCTCTTAGC	TTCCTTCAACTCTACTRCCCCC	–

^#^Human RSV N gene D00736 (GenBank, NCBI) used as reference sequence, *described in [6], **requires further validation and assessment.
